# Supplementary material for: Long-lived unipotent Blimp1-positive luminal stem cells drive mammary gland organogenesis throughout adult life
Source: Nat Commun. 2017 Nov 20;8:1714. doi: 10.1038/s41467-017-01971-w (PMC5696348; doi:10.1038/s41467-017-01971-w)
Supplement: Supplementary file 1 — Supplementary Information [file 41467_2017_1971_MOESM1_ESM.pdf]

Supplementary information

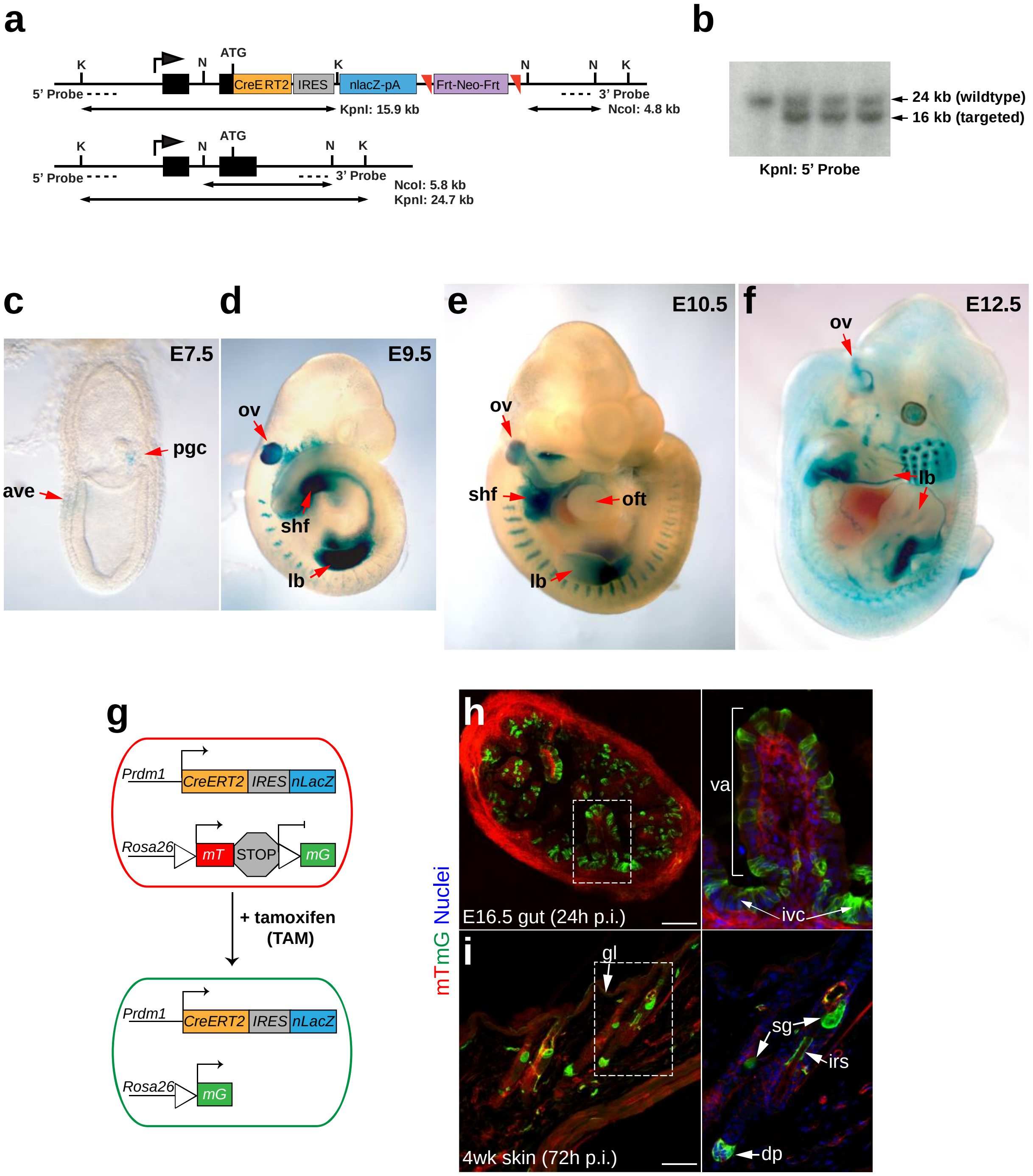

Supplementary Figure 1 Generation and validation of a dual-purpose *Prdm1.CreERT2-LacZ* reporter allele. (a) Targeting strategy for generating the *Prdm1-CreERT2-IRES-nlacZ* allele. Mutant (top) and wild-type (bottom) alleles with Southern blotting restriction enzyme sites, fragment sizes, and location of probes indicated. (K) KpnI; (N) NcoI; (ATG) Prdm1 translation initiation methionine codon. **(b)** Southern blot screening of embryonic stem (ES)

cell clones. **(c–f)** X-gal staining of *Prdm1Cre^{ERT2}-LacZ* embryos, to monitor endogenous *Prdm1* transcriptional activity. **(c)** At E7.5, PGCs are readily detectable. **(d)** E9.5 LacZ expression in the otic vesicle (ov), emerging forelimb bud (lb), and second heart field (shf) faithfully reflects previously documented sites of mRNA expression. *Prdm1Cre^{ERT2}-LacZ* reporter expression in E10.5 **(e)** and E12.5 **(f)** embryos shows down-regulation of transcriptional activity in the forelimb and shf. **(g)** Lineage tracing strategy. Intraperitoneal administration of tamoxifen (TAM) into *Prdm1Cre$^{ERT2/+}$;R26R$^{mTmG/+}$* females leads to nuclear accumulation of Cre recombinase and concomitant excision of the floxed reporter locus, switching expression of membrane-bound tdTomato (mT) to membrane-bound GFP (mG). **(h)** Cryosections (20 μm-thick) embryonic intestine analysed at 24h post-TAM (0.5 mg/25 g body weight) induction (p.i.) in pregnant females at pregnancy day (P) P15.5, stained for GFP (green) and counterstained with DAPI (Nuclei, blue). mT is displayed in red. After 24h GFP^{+} labelled cells are detected in the highly proliferative intervillous pocket cells (ivc), and forming villus axis (va) (*n*=3 embryos). Scale bars, 50 μm. **(i)** Cryosections (20 μm-thick) of adult postnatal skin analysed at 72h post-TAM (4 mg/25 g body weight) induction (p.i.), stained for GFP (green) and counterstained with DAPI (Nuclei, blue). mT is displayed in red. After 72h GFP^{+} labelled cells are detectable in the sebaceous gland (sg), dermal papillae (dp) and inner root sheath (irs) of the hair follicles as well as a subset of the granular layer (gl) keratinocytes (*n*=3 embryos). Scale bars, 50 μm.

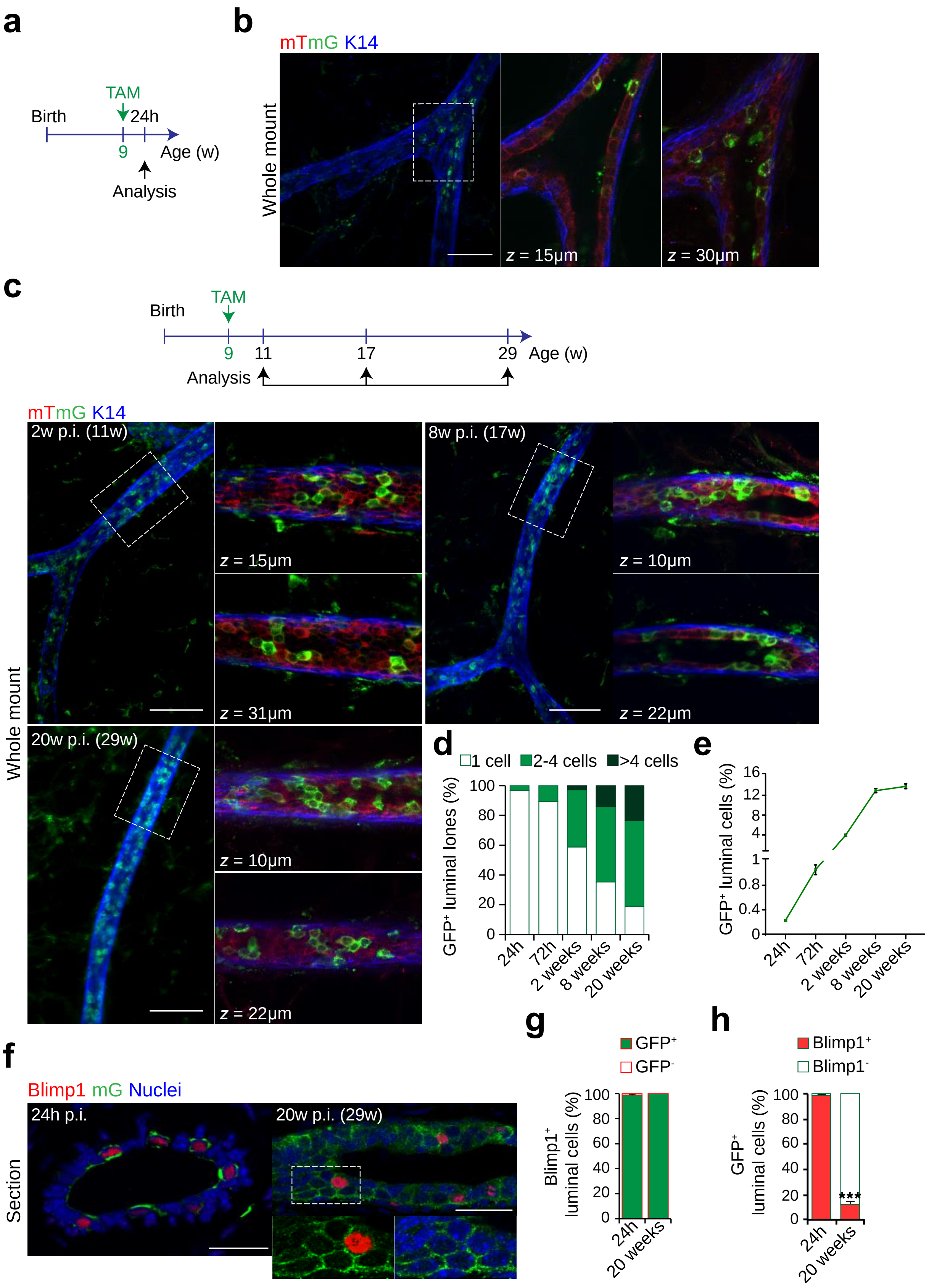

Supplementary Figure 2 Blimp1-derived cells represent luminal progenitors that contribute actively to ductal homeostasis. (a) Lineage tracing strategy adopted in **(b, d-h)**.

9-week-old adult virgin *Prdm1Cre*$^{ERT2/+}$;*R26R*$^{mTmG/+}$ females injected with TAM (4 mg/25 g body weight) were analysed 24h later. **(b)** 3D imaging of SeeDB-cleared of 9-week-old virgin mammary tissue. Glands are stained for GFP (green) and K14 (blue). mT is displayed in red. (*n*=3 mice). Scale bar, 100 µm. **(c)** Lineage tracing strategy adopted in **(c-h)**. 9-week-old adult virgin *Prdm1Cre*$^{ERT2/+}$;*R26R*$^{mTmG/+}$ females injected with TAM (4 mg/25 g body weight) were analysed at 2w, 8w or 20w post-induction with TAM (p.i.) at 11w, 17w or 29w after birth. 3D imaging of SeeDB-cleared of adult mammary tissue at 2w, 8w and 20w p.i. Glands are stained for GFP (green) and K14 (blue). mT is displayed in red. (*n*=4: 2w p.i.; *n*=3: 8w p.i.; *n*=3: 20w p.i.). Scale bars, 100 µm. **(d)** Percentages of GFP$^+$ clones containing 1, 2-4, and >4 cells at different time points post-TAM induction. Data are presented as mean ± s.e.m. (Number of clones pooled from *n*=3 mice per time point: *n*=322, *n*=347, *n*=438, *n*=456, *n*=513, respectively). ***$P<0.001$ (one-way ANOVA). **(e)** Percentages in adulthood of GFP$^+$ cells within the total luminal cell population, at different time points post-TAM induction. Data are presented as mean ± s.e.m. (Number of GFP+ cells pooled from n=3 mice per time point: *n*=730, *n*=1386, *n*=2691, *n*=4200, *n*=4509, respectively). ***P<0.001 (one-way ANOVA). **(f)** Cryosections (20 µm-thick) from adult mammary glands analysed at 24h and 20w post-TAM induction (p.i.), stained for GFP (green), Blimp1 (red) and counterstained with DAPI (Nuclei, blue). (*n*=3 mice per time point). Scale bars, 50 µm. **(g)** Percentages in adulthood of Blimp1$^+$GFP$^+$ *versus* Blimp1$^+$GFP$^-$ cells at 24h and 20w post-TAM induction. Data are presented as mean ± s.e.m. (Number of Blimp1$^+$ cells pooled from *n*=3 mice per time point: *n*=227 and *n*=209, respectively). $P>0.05$ (*t* test). **(h)** Percentages in adulthood of GFP$^+$Blimp1$^+$ versus GFP$^+$Blimp1$^-$ cells at 24h and 20w post-TAM induction in adulthood. Data are presented as mean ± s.e.m. (Number of GFP$^+$ cells pooled from *n*=3 mice per time point: *n*=234 and *n*=2157, respectively). ***$P<0.001$ (*t* test).

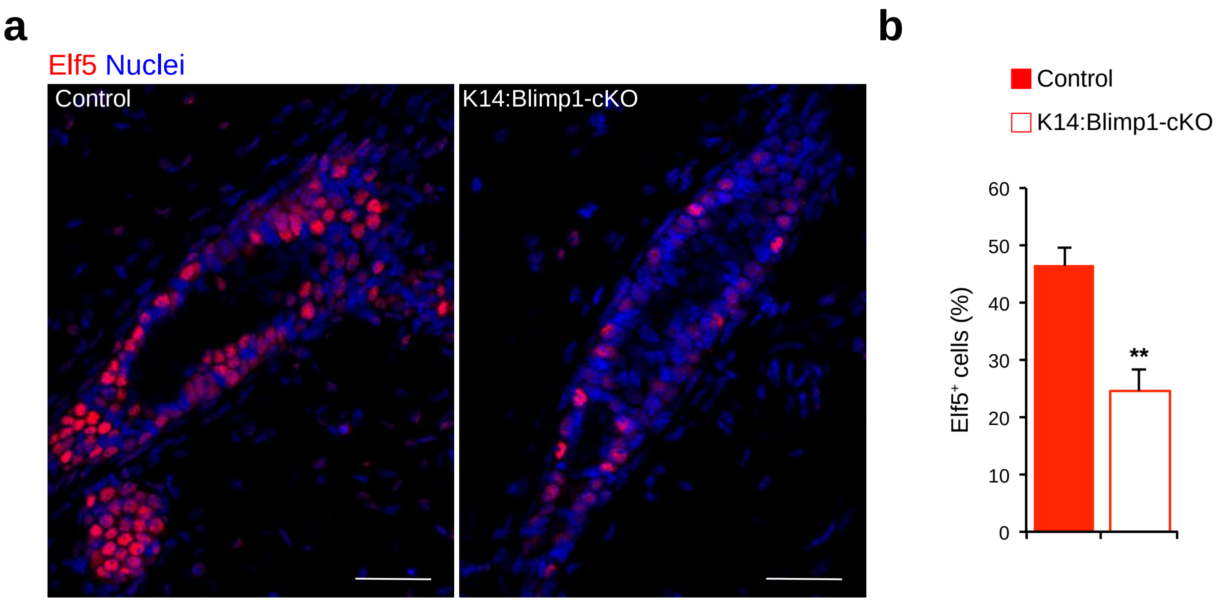

Supplementary Figure 3 Blimp1 conditional loss affects Elf5$^+$ luminal cell population. **(a)** Cryosections (20 μm-thick) from control and K14:Blimp1-cKO 11-week-old adult virgin mammary glands stained for Elf5 (red) and counterstained with DAPI (Nuclei, blue). (*n*=3 mice per genotype). Scale bars, 50 μm. **(b)** Percentage of Elf5$^+$ cells in control *versus* K14:Blimp1-cKO 11-week-old adult virgin mammary glands. Data are presented as mean ± s.e.m. (*n*=3 mice per genotype). $^{**}P<0.01$ (*t* test).

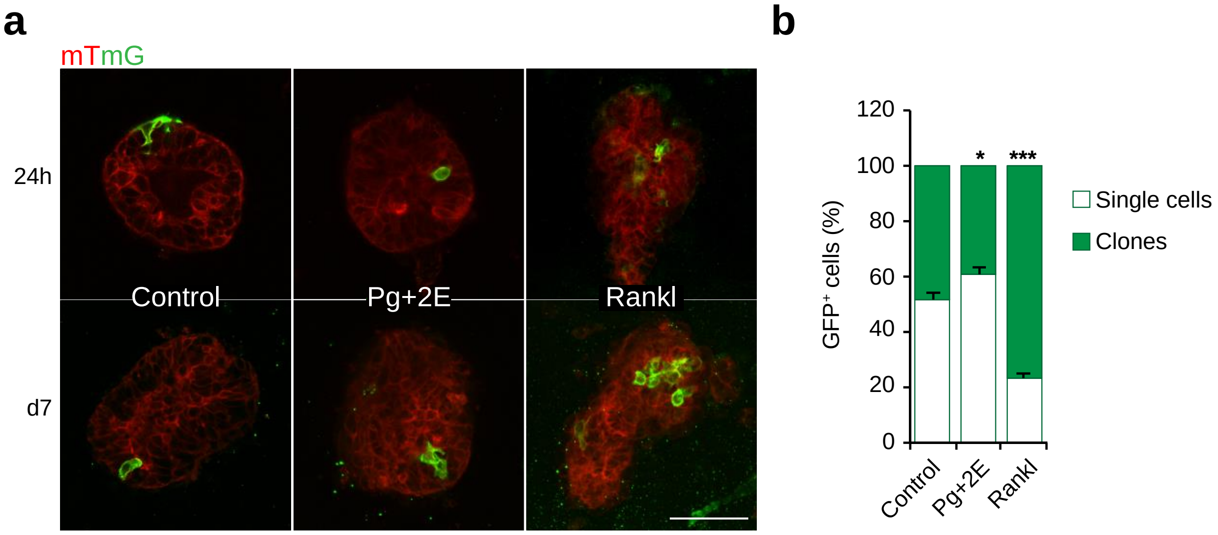

Supplementary Figure 4 Blimp1⁺ cells respond properly to pregnancy-induced signals. **(a)** 3D organoids from 11-week-old adult virgin *Prdm1Cre*$^{ERT2/+}$;*R26R*$^{mTmG/+}$ females were induced with 4-hydroxytamoxifen (4-OHT) *in vitro* and treated for 7 days (d7) with ethanol (Control), progesterone and 17β-estradiol (Pg+2E) or Rankl. mT^{+} cells are displayed in Red and GFP^{+} cells represent Blimp1-expressing progenitors at 24h and progeny at d7 (3 independent experiments, *n*=3 mice per experiment). Scale bars, 50 μm. **(b)** Quantification of clonal expansion of GFP^{+} cells at d7 in response to Pg+2E or Rankl. The percentages of counted GFP^{+} clones (Single cells *versus* Clones) containing 1, 2 or more cells, respectively, are displayed. Data are presented as mean ± s.e.m. from 3 independent experiments (*n*=3 mice per experiment). *$P<0.05$; ***$P<0.001$ (*t* test).

Supplementary Table 1 List of antibodies.

Antibody	Source	Catalogue number
Anti-Blimp1, rat monoclonal (Clone 6D3)	eBioscience	14-5963
Anti-GFP, chicken polyclonal	Abcam	ab13970
Anti-K8, guinea pig polyclonal	Progen	GP11
Anti-K14, rabbit polyclonal	Covance	PRB-155P
Anti-ERα, rabbit polyclonal	Santa Cruz	sc-542
Anti-PR, rabbit polyclonal	Abcam	ab131486
Anti-Elf5, goat polyclonal	Santa Cruz	sc-9647
Anti-Ki67, mouse monoclonal (Clone MM1)	Novacasta Laboratories	NCL-L-Ki67-MM1
APC conjugated anti-CD31, rat monoclonal (Clone MEC13.3)	BD Pharmingen	551262
APC conjugated anti-CD45, rat monoclonal (Clone 30-F11)	BD Pharmingen	559864
PerCP-Cy5.5 conjugated anti-CD24, rat monoclonal (Clone M1/69)	BD Pharmingen	562360
PE-Cy7 conjugated anti-CD49f, rat monoclonal (Clone GoH3)	Biolegend	313622
Alexa Fluor 488 conjugated goat anti-chicken IgG	Life Technologies	A11039
Alexa Fluor 647 conjugated donkey anti-guinea pig IgG	Life Technologies	A21450
Alexa Fluor 647 conjugated donkey anti-goat IgG	Life Technologies	A21447
Alexa Fluor 647 conjugated goat anti-rabbit IgG	Life Technologies	A21245
Alexa Fluor 633 conjugated goat anti-rat IgG	Life Technologies	A21094
Alexa Fluor 405 conjugated goat anti-rabbit IgG	Life Technologies	A31556
